# *Escherichia coli* vacuolating factor, involved in avian cellulitis, induces actin contraction and binds to cytoskeleton proteins in fibroblasts

**DOI:** 10.1590/1678-9199-JVATITD-2020-0106

**Published:** 2021-03-05

**Authors:** Annelize Zambon Barbosa Aragão, Natália Galdi Quel, Paulo Pinto Joazeiro, Tomomasa Yano

**Affiliations:** 1 Department of Genetics, Evolution, Microbiology and Immunology, Institute of Biology, State University of Campinas (Unicamp), Campinas, SP, Brazil.; 2 Department of Biochemistry and Tissue Biology, Institute of Biology, State University of Campinas (Unicamp), Campinas, SP, Brazil.

**Keywords:** Escherichia coli, *Escherichia coli* vacuolating factor (ECVF), Avian pathogenic *Escherichia coli* (APEC), Cytoskeleton

## Abstract

**Background:**

Avian pathogenic *Escherichia coli* (APEC) isolated from avian cellulitis lesions produces a toxin, named *Escherichia coli* vacuolating factor (ECVF), that causes cell vacuolization and induces inflammatory response in broiler chicken.

**Methods:**

We investigated the intracellular activities of ECVF in avian fibroblasts using fluorescence staining, electron microscopy, MTT and LDH measurements. As ECVF act specifically in avian cells, we performed blotting assay followed by mass spectrometry to better understand its initial intracellular protein recognition.

**Results:**

ECVF induced actin contraction, mitochondrial damage and membrane permeability alterations. Ultrastructural analysis showed intracellular alterations, as nuclear lobulation and the presence of degraded structures inside the vacuoles. Moreover, ECVF induced cell death in fibroblasts. ECVF-biotin associates to at least two proteins only in avian cell lysates: alpha-actinin 4 and vinculin, both involved in cytoskeleton structure.

**Conclusion:**

These findings demonstrated that ECVF plays an important role in avian cellulitis, markedly in initial steps of infection. Taken together, the results place this toxin as a target for drug and/or vaccine development, instead of the use of large amounts antibiotics.

## Background

Avian pathogenic *Escherichia coli* is a group of *E. coli* that cause extra-intestinal colibacillosis in poultry that is responsible for important worldwide economic losses [1-3]. APEC is also relevant to humans because it shares some similarities with human extra-intestinal pathogenic *E. coli* (ExPEC), which claims attention to a possible zoonotic risk [4-7]. The similarities in their genomic sequences, serogroups, antibiotic resistance and other abilities to cause disease suggest that APECs are a reservoir of virulence factors (VF) to ExPEC [6,8,9]. Adhesins, capsules, iron acquisition systems and secreted toxins [10] are VF implicated in avian diseases.

Previously, a heat-labile cytotoxin produced by an APEC isolated from avian cellulitis lesions was described inducing vacuolization only in avian cells [11], this cytotoxin was called ECVF. Parreira and Gyles [12] also described a pathogenicity island (PAI) adjacent to the thrW tRNA, which encode an autotransporter toxin, that was named vacuolating autotransporter toxin (Vat). Furthermore, cellular vacuolization induced by Vat and ECVF are similar to VacA, a cytotoxin produced by *Helicobacter pylori* [13]. Although VacA is specific for mammalian cells [14], ECVF is toxic only to avian cells [11].

Later, Quel et al. [15] observed that purified ECVF induces acute inflammatory response in the epidermis, dermis and panniculus of broiler chickens, similar to cellulitis inflammation induced by *E. coli* AC53 (isolated from cellulitis lesions). However, the molecular action of the toxin in the avian cells has not yet been clarified. In this study, we proposed to better understand the multiple biological damage that ECVF induces in avian cells, leading them to death. We believe that ECVF could be a potential target to drug development against cellulitis development in poultry.

## Methods

### ECVF production and purification

ECVF was produced by *E. coli* AC 53 (serogroup O21,83) kindly provided by Dr. Carlton Gyles (University of Guelph, Ontario, Canada), isolated from cellulitis lesions [16]. The toxin was produced and purified as described previously [15]. After purification, ECVF toxin concentration was measured using Bio-Rad Protein Assay and the cytotoxic dose for 50% of viability (CD_50_) was determined at 40 µg mL^-1^ (data not shown).

### Cell culture

In this study we used two avian fibroblasts: a primary chicken embryo fibroblasts (CEF), kindly prepared and donated by Fort Dodge Animal Health (Campinas, São Paulo, Brazil) and CEC-32, that was kindly provided by Dr. Fabiana Horn (Biosciences Institute of Federal University of Rio Grande do Sul, Porto Alegre, Rio Grande do Sul, Brazil). Both cells were maintained at Eagle´s minimal essential medium (EMEM - Nutricell) supplemented with 10% fetal bovine serum (FBS - Sigma) and 1% of an antibiotic solution (100,000 U/L penicillin and 10 mg/L streptomycin - Sigma). To all experiments, the controls were treated with 60mM Tris-HCl pH7.4, which was the purification buffer, in the same volume that was applied in the ECVF-treated cells. Additionally, HUVEC and Vero cells were cultured at the same conditions as described above, until the use in ECVF-biotin assay.

### Fluorescence staining

*Fluorescence actin staining (FAS)*

FAS was performed as previously described [17]. Fibroblasts were incubated with purified ECVF (2.5 × CD_50_) and observed during 24 h**.** After incubation, avian cells were fixed with 2% formalin, washed, permeabilized by adding 0.1% Triton X-100, and stained with 0.05 mg/mL of phalloidin-TRITC (Sigma) in PBS. CEC-32 nuclei were additionally stained with 4’,6-diamidino-2-phenylindole (DAPI). Slides were mounted with 90% glycerol, covered with a glass cover slide, and examined at a fluorescence microscope (LEICA DM 2500).

*Acridine orange (AO)*

Dye accumulation was assessed by exposing living CEF cells, after vacuolization induced by exposure to ECVF (CD_50_) for 6, 18 and 24 h, to 5 μg/mL of AO (Sigma) for 2 min in EMEM as described by Catrenich and Chestnut [18]. Cover-slips bearing cells were then rinsed three times in PBS, mounted on microscope slides and observed immediately (LEICA DM 2500). All observations were recorded until 10-15 min after AO exposure, to retain the cell morphology.

*Transmission electron microscopy (TEM)*

To exploit the endomembrane system under effects of ECVF, vacuolization was induced in CEC-32, which were observed using TEM. The avian fibroblasts (2 x 10^6^ cells/mL) were treated with purified toxin (CD_50_), at different times: 2, 4, 6, 18 and 24 hours. Cells were fixed for 1h at RT with 2% glutaraldehyde in 0.1 M sodium cacodylate buffer (pH 7.4), post-fixed with 1% osmium tetroxide for 1h at 4°C and, subsequently with 1% uranyl acetate for 15 min at 4°C. The avian cells were then dehydrated in ascending concentrations of ethanol and embedded in Epon resin (Polysciences Inc.). Ultrathin sections were stained with 0.5% lead citrate and examined in a transmission electron microscope (LEO-906) at 60kV.

### Cytotoxicity and cell death monitoring

*MTT assay*

The mitochondrial viability assay [19], detects the cellular reduction of 3-(4,5-dimethylthiazol-2-yl)-2,5-diphenyltetrazolium bromide (MTT) to formazan, by the mitochondrial dehydrogenase. Briefly, CEF cells were plated in 96-well plate (2 x 10^4^ cells/mL) for 24 h and treated with ECVF (CD_50_) for 2, 4, 6, 15, 24, 36 and 48h. Cells were washed and fresh medium containing MTT solution (0.8 mg/mL) was added to each well. After 3 h at 37°C, in the dark, the medium was removed and the formazan was solubilized in HCl 1N - isopropanol (1:24 v/v). The plates were shaken for 10 min and the absorbance measured at 550 nm (reference to 700 nm). Two independent experiments were performed in triplicates.

*Lactate dehydrogenase (LDH) release assay*

Changes in cell membrane integrity of CEF exposed to ECVF (CD_50_) was determined as previously described [20]. Briefly, CEF cells (2 x 10^4^ cells/mL) were treated with ECVF at different time intervals (2, 4, 6 and 12h), the supernatant was collected and centrifuged at 400 × g per 5 min at 4°C (UniCenMR Herolab). An aliquot of 0.1 mL of the supernatant was placed in a cuvette containing 2.5 mL of phosphate buffer at 37°C combined with 0.2 mL of NADH (2.5 mg/mL) and 0.2 mL of sodium pyruvate (1 mg/mL). Kinect was measured at intervals of 30s until reach 3 min (end point) at 340 nm. CEF cells exposed to 10% SDS was used as a positive control for total LDH release.

*DNA fragmentation detection by terminal deoxynucleotidyl transferase dUTP nick end labeling (TUNEL)*

To better understand the cell death process induced by ECVF, CEC-32 cells (2 × 10^6^ cells/mL) were treated with the toxin (CD_50_), at different times: 2, 4, 6, 18 and 24 hours. Cells were fixed with 1.5% paraformaldehyde in PBS for 1h at 4°C. After that, fibroblasts were rinsed with PBS and subsequently permeabilized (0.1% sodium citrate and 0.1% Triton X-100) for 2 minutes at 4°C. Then, cells were incubated with the commercially available In Situ Cell Death Detection Kit (Roche Molecular Biochemicals), following the manufacturer´s instructions. After counter-stained with DAPI, slides were mounted with 80% glycerol and observed immediately at fluorescence microscope (LEICA DM 2500). Total and death cells were counted in 5 different fields per condition and the results were subjected to a statistical analysis using GraphPad Prism, version 5.

### Host proteins recognition by ECVF

*ECVF biotinylation*

Lyophilized ECVF was resuspended in PBS containing 10 mg mL^-1^ of biotin (EZ-link sulfo-NHS-LC-biotin - Thermo Fisher Scientific) and incubated for 1 hour at 37°C. Then, the excess of biotin was removed in a desalting column (ZebaTM Spin Desalting Column - Thermo Fisher Scientific) and biotinylated ECVF was used to detect proteins in a blotting assay according to the methodology below. The entire ECVF biotinylation process was performed according to the manufacturer's recommendations.

*Interaction with ECVF-biotin and host cell proteins*

Cell monolayers (HUVEC, CEF and Vero) were washed twice with sterile PBS, pH 7.4, and resuspended by scraping in the same buffer. Cell disruption was made using ultrasound (amplitude of 20% for 2 min.). Then, the insoluble pellet was removed by centrifugation (10000 × *g* for 15 min at 4 °C) and the supernatant was used as described below. Protein dosage was performed as described by Bradford [21]. SDS-PAGE was performed according to the methodology proposed by Laemmli [22]. Protein extracts (50 µg for each cell line) were combined with the sample buffer (v/v) and boiled for 5 min. Proteins separation was carried out with 2 gels, one was stained by Coomassie bright blue (R 250) and the other was used for western blotting (performed as described by Renesto et al. [23], with modifications). Proteins separated in the SDS-PAGE were transferred to nitrocellulose membrane (Bio-rad), subsequently blocked in the TBS-T solution (Tris buffer, supplemented with 0.1% Tween 20 and 5% BSA) for 1 h, at 4 °C. Then, the membrane was incubated in the TBS-T solution containing ECVF-biotin, in the proportion of 1: 200 (v/v), overnight, at 4 °C. Membranes were washed three times with TBS-T and incubated with peroxidase-conjugated streptavidin (1:1000, GE Healthcare). The detection of ECVF binding to cellular proteins was revealed by chemiluminescence.

*Protein identification by mass spectrometry (MS)*

In order to identify the proteins that compose the interaction with the ECVF, the band was analyzed by mass spectrometry, performed by CEFAP (Center for Research Facilities, ICB-USP) in a LTQ Orbitrap Velos (Thermo Fisher). Raw data was processed in Mascot against *Gallus gallus* database.

## Results

### ECVF induces contraction of the actin filaments

**Figure 1.**
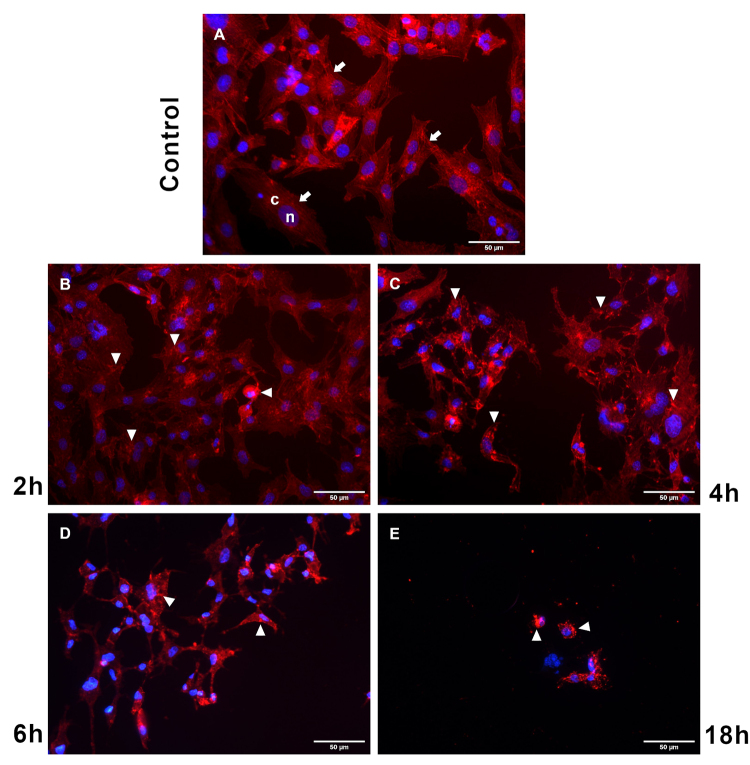
Alterations in cell morphology due to the contraction of the actin filaments. **(A)** CEC-32 cells without ECVF, 24 hours. **(B, C, D** and **E)** CEC-32 after ECVF incubation for 2, 4, 6, and 18 hours, respectively. Red: Phalloidin-TRITC; blue: DAPI. Arrows indicate normal actin filaments and arrowheads indicate contracted actin filaments. Scale bars = 50 μm.

CEC-32 cells were treated with high amounts of ECVF toxin (2.5 × CD_50_). After only 2 hours of incubation (Figure 1B), clearly changes in cell morphology were observed, such as cytoplasmic alterations followed by nuclear and chromatin modifications. Actin was under retraction and it was possible to observe a consequent loss of adhesion to the cell support and neighboring cells, leading to monolayer disruption (Figure 1). These effects were more evident after 18h (Figure 1E) and 24h (data not shown) of exposure to ECVF, when progressive loss of nuclei per field was observed. CEF cells, treated with a lower concentration of ECVF, also showed changes in the cytoskeleton compared to control cells. After incubation for 6 hours, some of these primary fibroblasts were rounded, due to the contraction of the actin filaments (Additional file 1).

### AO uptake indicates cytoplasmic acidification caused by ECVF

**Figure 2.**
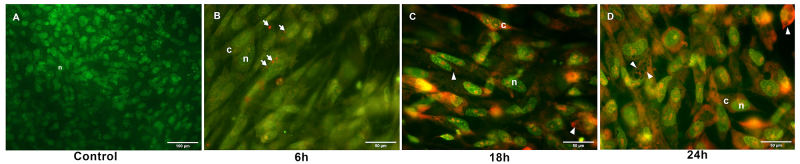
AO pattern changes inside CEF cells after ECVF treatment. **(A)** Control without ECVF, 24 hours. **(B, C** and **D)** CEF after ECVF incubation for 6, 18 and 24 hours, respectively. n: nucleus; c: cytoplasm. Arrows indicate lysosomes in orange and arrowheads indicate unstained vacuoles. Scale bars = 100 μm **(A)**; 50 μm **(B, C** and **D)**.

CEF cells were treated with ECVF toxin during 6, 18 and 24h and then were stained with AO, a fluorescent probe used to follow the pH changes in vacuoles or acid vesicles. Control cells showed only the nucleus stained in green (Figure 2A), as expected. After the first 6h in contact with ECVF toxin, bright orange spots appeared in the cytosol (Figure 2B), probably due to AO protonation and accumulation into the acid lysosomes. ECVF-induced damage is time-dependent, as after 18h it was possible to observe a loss in the acid pH compartmentalization, since the entire cell appears diffusely yellow-orange fluorescent (Figure 2C and D). After 24h, the vacuoles induced by ECVF remain unstained, suggesting that these compartments are non-acid (Figure 2D).

### ECVF induces nuclear and cytoplasmic alterations

**Figure 3.**
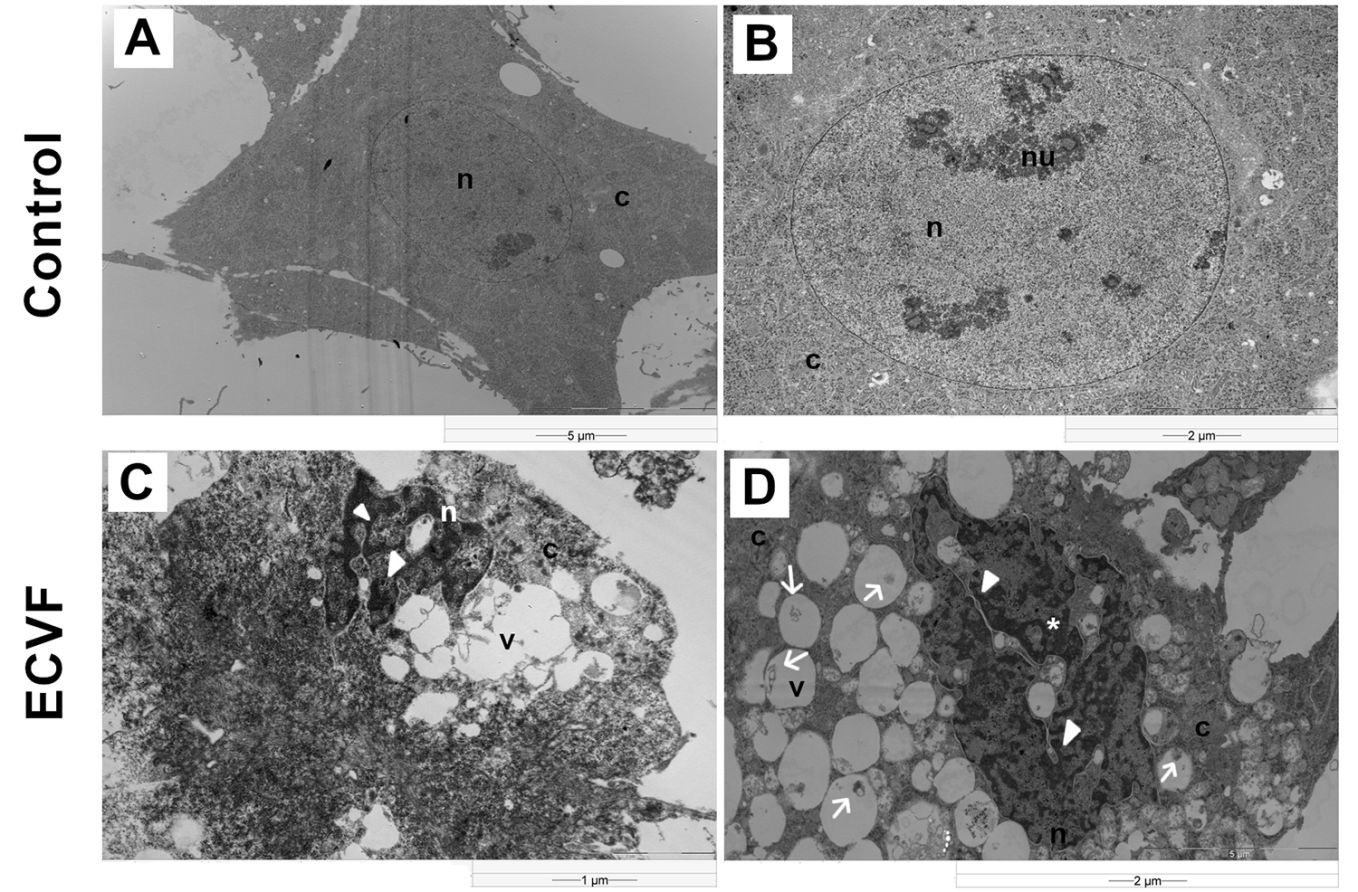
Intracellular alterations in CEC-32 fibroblasts induced by ECVF treatment. **(A, B)** Control, untreated fibroblasts, after 24 hours. Electron micrographs of untreated cells show endomembrane system distributed in a less compact cytoplasm and a round-shaped nucleus with orderly euchromatin, typical of nucleus at the interphase. **(C, D)** Electron micrographs of treated cells show endomembrane system with numerous vacuoles of irregularly shapes, filled with membranous components. Vacuoles also show signs of swelling and are distributed in compacted cytoplasm. Nuclei exhibit irregular shape and display an abundance of lobulations and also a high content of heterochromatin associated to nuclear envelope. n: nucleus; nu: nucleolus; c: cytoplasm; v: vacuoles. *Asterisks indicate chromatin condensation, arrowheads indicate nuclear lobulation and arrows indicate intravacuolar structures. Scale bars = 5 μm **(A)**, 2 μm **(B, D)**, 1 μm **(C)**.

The control cell has an intact nucleus, well-organized nucleoli and lightly packed chromatin (Figure 3A and B). On treated cells, ECVF induced intense cytoplasmic vacuolization, along with disorganization of the endomembrane system, mitochondria as well as cytoskeleton, when compared with untreated cells. After 6 hours of treatment, it was possible to see some chromatin condensation within the nucleus, nuclear shrinkage and apoptotic bodies; moreover, these effects became more pronounced with time (Figure 3C). In addition, after 18 hours of exposure to ECVF, it was possible to observe membranous components inside the vacuoles (Figure 3D).

### Mitochondrial activity and plasma membrane integrity are affected

**Figure 4.**
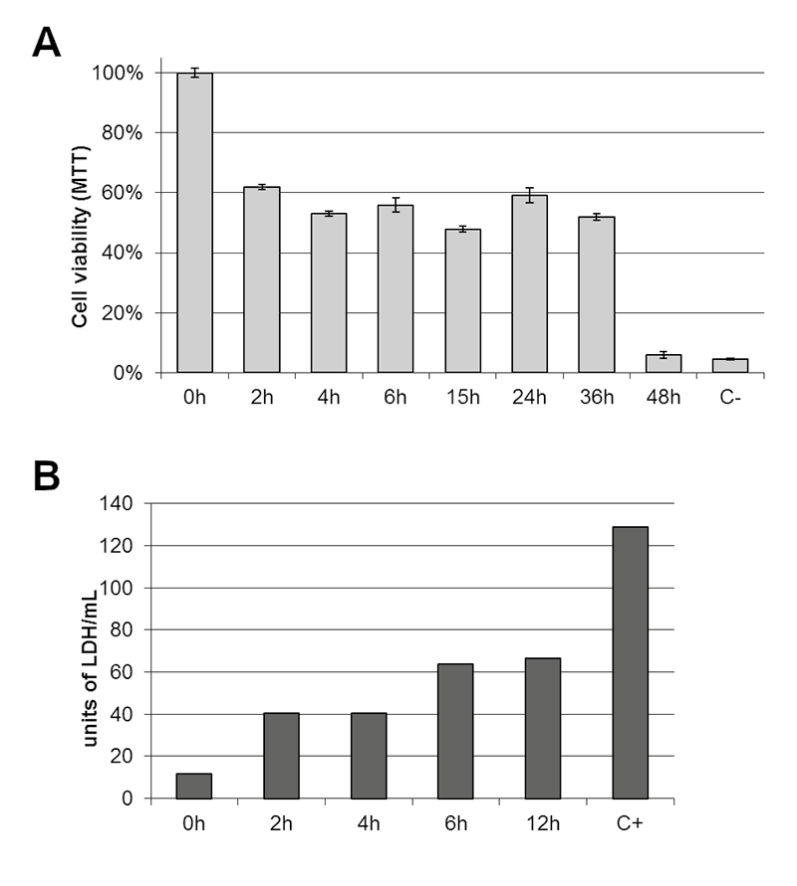
ECVF affects the mitochondrial activity and causes injury in membrane integrity in CEF cells. **(A)** MTT assay shows that CEF viability decreases to 60% after 2 hours of incubation with ECVF. Performed in triplicates. **(B)** Membrane integrity was lost and LDH had a maximum release after 6 hours of ECVF incubation (C+ represents the total LDH release after SDS treatment).

CEF cells were evaluated for mitochondrial viability and membrane integrity. Results of mitochondrial viability are shown in Figure 4A. Cell viability was affected since the first 2 hours after ECVF inoculation, and the mitochondrial activity was reduced to less than 60% after 24 hours. Membrane integrity of CEF cells was assessed by LDH release assay. Membrane injury started at 2 hours after ECVF treatment. After 6 hours, the maximum release of the LDH enzyme was observed (Figure 4B).

### Fibroblast death was detected by TUNEL

**Figure 5.**
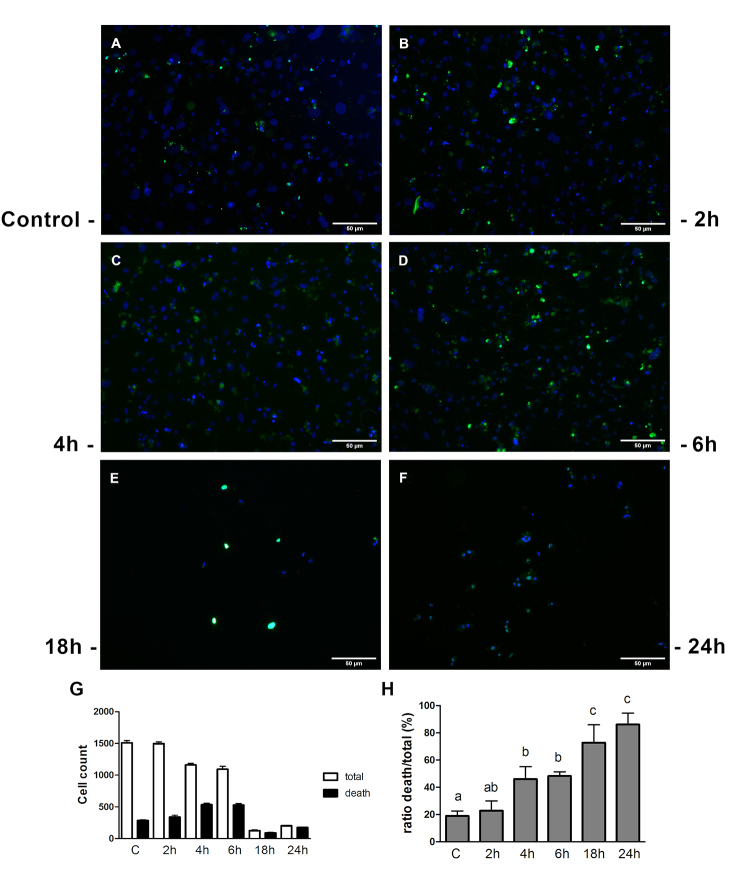
ECVF induces death in avian fibroblasts. **(A)** CEC-32 control, 24 hours. **(B, C, D, E** and **F)** CEC-32 after ECVF exposure, assayed using TUNEL. 2, 4, 6, 18 and 24 hours, respectively. Representative fields of each condition. **(G)** Total and death cells were counted for each time of ECVF treatment in 5 different fields. Bars represent the sum of these 5 fields. **(H)** Percentage of the ratio between death/total cells (a, b and c represent statistical different groups). ANOVA one-way, followed by Tukey’s test.

The TUNEL assay, performed in CEC-32 cells, showed classic condensation and fragmentation of nuclei (Figure 5). The quantity of TUNEL-positive cells is represented in Figure 5A and 5B. The significant number of labeled cells in this assay was difficult to assess since we noticed a marked decrease in the number of cells, which became detached from the cover slips, after 18-24 hours of incubation with the toxin.

### ECVF recognizes only avian proteins

**Figure 6.**
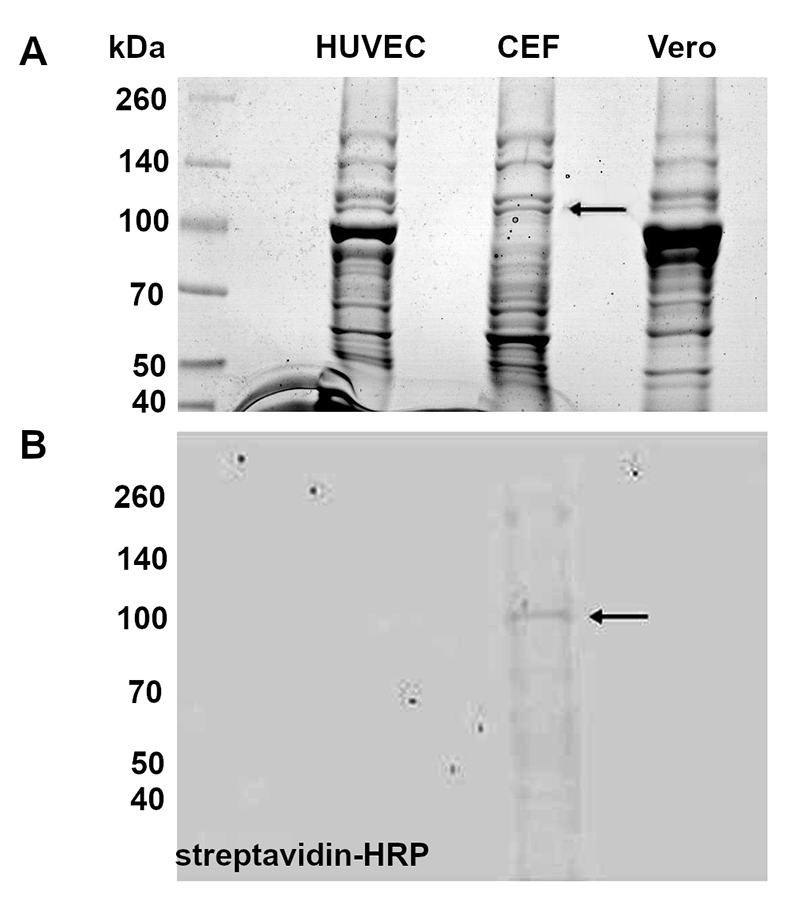
Detection of avian proteins, possibly involved in the effects of ECVF on cells. **(A)** SDS-PAGE showing the proteins extracted from HUVEC, CEF and Vero cells. **(B)** ECVF-biotin incubated membrane was revealed using peroxidase-conjugated streptavidin. Arrow indicates the CEF’s proteins band for which interaction with ECVF was detected.

Western blotting demonstrated that the ECVF-biotin shows specificity to recognize proteins of avian cells (Figure 6), since there was only interaction with CEF's proteins, and no signal was detected for the proteins extracted from mammalian cell lines (HUVEC and Vero).

## Discussion

Many poultry diseases are caused by *E. coli*, including the yolk sac infection syndrome, head-swelling, septicemia and cellulitis. Avian cellulitis is asymptomatic in the living bird and it is found only after slaughter, causing important economic losses. *E. coli* have been commonly isolated from avian cellulitis lesions [24-26] and its relevance in this disease has been suggested by several authors [15,27-29]. Moreover, the presence of aerobactin, fimbriae and cytotoxins may increase the ability of *E. coli* to colonize the subcutaneous tissues of chicken [30].

Other bacteria are also capable to produce vacuolating cytotoxins. *H. pylori*, *Vibrio cholera* and *Aeromonas veronii* biovar *sobria*, produce toxins as VacA [31], a hemolysin [32] and VCF [33], respectively. In addition to the characteristic vacuolization previously described, in this study we took advantage of microscopy and *in vitro* assays to clarify how ECVF induces changes in avian fibroblasts.

Cytotoxins can work by modulating host cells to promote colonization and maintenance of the bacteria in host tissues. According to Cover and Blanke [14] bacteria that produce multifunctional toxins can use a single protein to obtain a range of actions in different tissues and host cells, enabling these pathogens to remodel tissues, allowing them to escape the host immune system defenses. Our results indicate that ECVF is a multifunctional toxin, damaging cell membrane, cytoplasm, organelles and nucleus.

Cytoskeleton alterations could be accessed using actin stained by labeled Phalloidin. Both CEC-32 and CEF cells show retraction of actin filaments when treated with ECVF. After 18h of cytotoxin exposure, fibroblasts had an intense contraction of the actin cytoskeleton, which had led to loss of adherence with the cell support and with neighboring cells (Figure 1), disrupting the cell monolayer. Noteworthy, cytoskeleton retraction is an important event during the early stages of apoptosis [34,35] and it could also contribute to a nuclear response via mechanoregulation (see below). Another alteration observed during ECVF treatment in living CEF cells, is related to the vacuole fluorescence. Vacuoles induced by VacA are strongly stained by AO [18], although ECVF’s vacuoles are unstained. Moreover, it is possible to see an evolution during the time course, as after the initial 6h, few orange spots are observed (Figure 2B), probably due to the AO penetration on lysosomes, full of acid content. However, after 18h and 24h the whole cytoplasm becomes red orange (Figure 2), indicating a loss in compartmentalization of these acid organelles.

To better understand what was going on inside the cells, CEC-32 were incubated with ECVF and observed by electron microscopy**.** It was possible to detect the presence of structures inside the vacuoles (Figure 3D). Similar structures were observed by Catrenich and Chestnut [18] in vacuoles induced by VacA in HeLa cells. These authors suggested that these structures are degraded cytoplasmic components, suggesting that the vacuoles are, actually, autophagosomes.

Our results for the MTT test are similar to previously described for HeLa cells and VacA toxin [36]. VacA requested more time for loss in mitochondrial activity than to the appearance of vacuoles. As well, here we showed that most fibroblasts (over 50%, data not shown) were vacuolated after 6 hours, while the acute loss on mitochondrial viability occurred after 36 hours of ECVF incubation (Figure 4A). Willhite et al. [36], demonstrated that the development of mitochondrial alterations in response to intracellular actions of VacA, caused cytochrome c release and activation of pro-apoptotic caspases. Likewise, our data shows that cytoplasmic membrane of CEF cells lost integrity, releasing approximately 67 units of LDH.mL^-1^, at 12 hours of treatment (Figure 4B). This means that ECVF induces several damages in avian fibroblasts, increasing cell death over time.

In addition to the evidences mentioned above, we used *In situ* detection of fragmented DNA (TUNEL assay) to confirm the cell death process induced by ECVF. Furthermore, electron micrograph showed a significant nuclear disorganization, with euchromatin condensation and nuclear lobulation (Figure 3C-D), whilst remarkably DNA fragmentation was confirmed by TUNEL (Figure 5A and 5B). Some authors [37-39] reported the relationship between apoptosis and tissue damage, suggesting that this type of cell death may be involved in the pathogenesis of some diseases caused by bacteria. VacA-induced cell death has been classiﬁed as an apoptotic process by several authors [40-42]. However, it was later demonstrated that VacA, in fact, induced a programmed necrosis in gastric epithelial cells [43]. Indeed, apoptosis and programmed necrosis, share mitochondrial-induced alterations and DNA damage, which were both observed in this study. As a matter of fact, mitochondria, play a central role in both modes of induced cell death, despite different cascades needs to be activated to lead the cell to death by either programmed necrosis or apoptosis [44,45]. ECVF induces death in avian fibroblasts, albeit the pathway by which it occurs remains to be elucidated.

The specificity between toxins and their host cells is extremely important to understanding the mechanisms involved in bacterial pathogenicity. According to Sewald et al. [46], VacA binds to specific receptors present in human cells that allows the toxin to bind to the cytoplasmic membrane, to be internalized and translocated, promoting vacuolization. Considering that ECVF could have a biological activity similar to that of VacA, it is reasonable to suggest that ECVF also has a specific receptor in avian cells that needed to be investigated. Hence, in this study, cytoplasmic proteins were identified in CEF (Figure 6), possibly involved with the intracellular damage caused by the toxin (Table 1). ECVF associates to alpha-actinin 4 and vinculin, both involved in cytoskeleton structure and maintenance. In this context, it is possible that ECVF acts on the nucleus via mechanoregulation, even before it is internalized. As reviewed by Hah and Kim [47], the nucleus can be regulated via changes in the cytoskeleton (by mechanosensing and mechanotransduction). Alpha-actinin is described as anchoring actin to a variety of intracellular structures and it is probably associated to vesicular trafficking [48-50]. Vinculin, is an F-actin binding protein involved in cell-matrix adhesion and cell-cell adhesion [51]. Although chicken alpha-actinin 4 and vinculin are very similar to their human orthologs (more than 90% of identity), ECVF was able to bind only to proteins presents in the avian cell lysate, suggesting that these protein-protein interactions could be important to the early stages of cellulitis infection and internalization of ECVF. This hypothesis is supported by the fact that this toxin does not induce vacuolization in mammalian cells [11], indicating that ECVF has a species-specific mode of action. Our results pave the way for deciphering the complete mechanism by which ECVF helps the bacteria to invade the avian cell and open the possibility to explore the relevance of ECVF in the pathogenesis of avian cellulitis, by future screening studies to deciphering which gene is responsible for encoding this toxin.

**Table 1.** List of the proteins identified by MS, probably involved in ECVF pathogenicity.

UniProt access	Description	Coverage (%)	#Unique pep	#Peptides
Q90734	**Alpha-actinin-4**	52,43	9	41
P12003	**Vinculin**	3,52	1	2

## Conclusion

In summary, our data confirms that ECVF is a multifunctional toxin, which causes a range of damages, culminating in cell death. The cytoskeleton disorganization and morphological changes induced by ECVF may play an important role in the initial steps of bacterial infection in avian cellulitis. Therefore, we consider that the ECVF toxin could be a target to drugs and/or vaccines against cellulitis infection in poultry, alternatively to the use of antibiotics.

### Abbreviations

AO: acridine orange; APEC: avian pathogenic *E. coli*; BSA: bovine serum albumin; CEC-32: chicken embryo cell-32; CEF: chicken embryo fibroblast; DAPI: 4’,6-diamidino-2-phenylindole; ECVF: *E. coli* vacuolating factor; EMEM: Eagle´s minimal essential medium; ExPEC: extra intestinal pathogenic *E. coli*; FBS: fetal bovine serum; FITC: fluorescein-5-isothiocyanate; HUVEC: human umbilical vein endothelial cells; LDH: lactate dehydrogenase; MS: mass spectrometry; MTT: 3-(4,5-dimethylthiazol-2-yl)-2,5-diphenyltetrazolium bromide; PAI: pathogenicity island; PBS: phosphate buffer saline; PI: propidium iodide; RT: room temperature; SDS: sodium dodecyl sulfate; SDS-PAGE: sodium dodecyl sulfate and polyacrylamide gel electrophoresis; TEM: transmission electron microscopy; TRITC: tetramethylrhodamine B isothiocyanate; TUNEL: terminal deoxynucleotidyl transferase dUTP nick end labeling; VacA: vacuolating cytotoxin A; Vat: vacuolating autotransporter toxin; VF: virulence factor.

## Supplementary material

The following online material is available for this article:

Additional file 1. CEF cells showing actin stained in green (Phalloidin-FITC). After 6h of ECVF treatment, an increased number of round shaped cells were observed.
