# Supplementary material for: Escherichia coli vacuolating factor, involved in avian cellulitis, induces actin contraction and binds to cytoskeleton proteins in fibroblasts
Source: J Venom Anim Toxins Incl Trop Dis. 2021 Mar 5;27:e20200106. doi: 10.1590/1678-9199-JVATITD-2020-0106 (PMC7941731; doi:10.1590/1678-9199-JVATITD-2020-0106)

J Venom Anim Toxins incl Trop Dis
https://doi.org/10.1590/1678-9199-JVATITD-2020-0106

Supplementary Material to "*Escherichia coli* vacuolating factor, involved in avian cellulitis, induces actin contraction and binds to cytoskeleton proteins in fibroblasts"

Additional file 1. CEF cells showing actin stained in green (Phalloidin-FITC). After 6h of ECVF treatment, an increased number of round shaped cells were observed (right side). Scale bars = 50 µm.

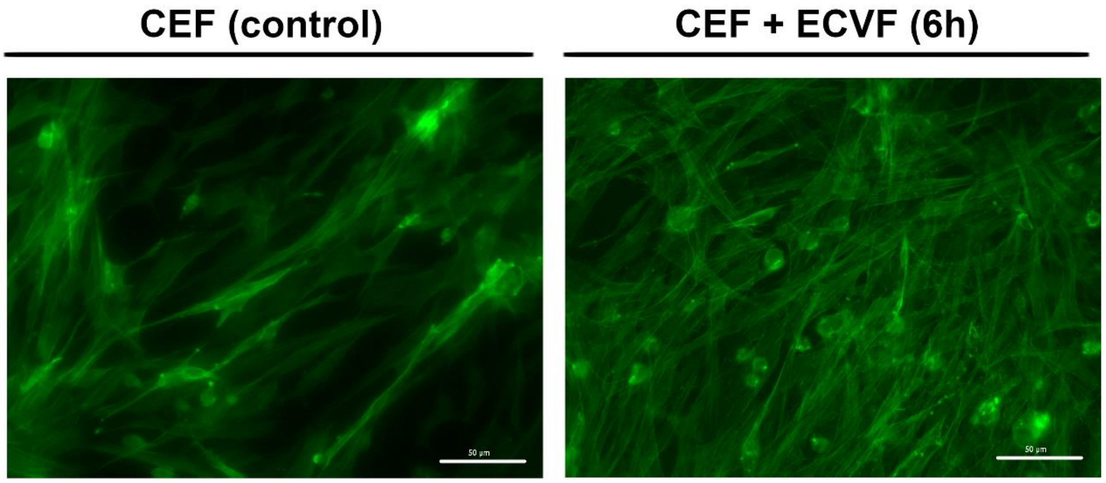

Supplement: Additional file 1. [file 1678-9199-jvatitd-27-e20200106-s1.pdf]
